# A 5-bp Insertion in *Mip* Causes Recessive Congenital Cataract in KFRS4/Kyo Rats

**DOI:** 10.1371/journal.pone.0050737

**Published:** 2012-11-30

**Authors:** Kei Watanabe, Kenta Wada, Tomoko Ohashi, Saki Okubo, Kensuke Takekuma, Ryoichi Hashizume, Jun-Ichi Hayashi, Tadao Serikawa, Takashi Kuramoto, Yoshiaki Kikkawa

**Affiliations:** 1 Mammalian Genetics Project, Tokyo Metropolitan Institute of Medical Science, Tokyo, Japan; 2 Graduate School of Life and Environmental Sciences, University of Tsukuba, Tsukuba, Japan; 3 Department of Bioproduction, Tokyo University of Agriculture, Abashiri, Japan; 4 Institute of Laboratory Animals, Graduate School of Medicine, Kyoto University, Kyoto, Japan; University of Arkansas for Medical Sciences, United States of America

## Abstract

We discovered a new cataract mutation, *kfrs4*, in the Kyoto Fancy Rat Stock (KFRS) background. Within 1 month of birth, all *kfrs4/kfrs4* homozygotes developed cataracts, with severe opacity in the nuclei of the lens. In contrast, no opacity was observed in the *kfrs4/*+ heterozygotes. We continued to observe these rats until they reached 1 year of age and found that cataractogenesis did not occur in *kfrs4/*+ rats. To define the histological defects in the lenses of *kfrs4* rats, sections of the eyes of these rats were prepared. Although the lenses of *kfrs4/kfrs4* homozygotes showed severely disorganised fibres and vacuolation, the lenses of *kfrs4/*+ heterozygotes appeared normal and similar to those of wild-type rats. We used positional cloning to identify the *kfrs4* mutation. The mutation was mapped to an approximately 9.7-Mb region on chromosome 7, which contains the *Mip* gene. This gene is responsible for a dominant form of cataract in humans and mice. Sequence analysis of the mutant-derived *Mip* gene identified a 5-bp insertion. This insertion is predicted to inactivate the MIP protein, as it produces a frameshift that results in the synthesis of 6 novel amino acid residues and a truncated protein that lacks 136 amino acids in the C-terminal region, and no MIP immunoreactivity was observed in the lens fibre cells of *kfrs4/kfrs4* homozygous rats using an antibody that recognises the C- and N-terminus of MIP. In addition, the *kfrs4/*+ heterozygotes showed reduced expression of *Mip* mRNA and MIP protein and the *kfrs4/kfrs4* homozygotes showed no expression in the lens. These results indicate that the *kfrs4* mutation conveys a loss-of-function, which leads to functional inactivation though the degradation of *Mip* mRNA by an mRNA decay mechanism. Therefore, the *kfrs4* rat represents the first characterised rat model with a recessive mutation in the *Mip* gene.

## Introduction

Kyoto Fancy Rat Stock (KFRS) strains are inbred strains derived from fancy rats to collect new rat mutations and increase the value of the rat model system. The founder rats are six fancy rats imported to Kyoto University from a fancy rat colony in the USA and six inbred lines (KFRS2/Kyo, KFRS3A/Kyo, KFRS3B/Kyo, KFRS4/Kyo, KFRS5A/Kyo, and KFRS6/Kyo), including two sublines that were produced by brother-sister mating after the initial cross with a laboratory strain, TM/Kyo or PVG/Seac. The KFRS strains are a potential source of novel rat mutations because mutations, such as those affecting coat and eye colour, occur frequently in fancy rats. Indeed, we have identified 16 mutations that affect coat colour, eye colour, and hair pattern in the KFRS strains [1]. In addition, fancy rat colonies are thought to have been maintained relatively independently of laboratory rats [2]. This characteristic suggests that fancy rats have a unique genetic background that is more similar to that of rat strains that were recently derived from wild rats than to that of laboratory rats; therefore, KFRS strains are likely to become a new powerful tool for forward genetic studies of various pathogenic phenotypes among human populations and for providing valuable biological information regarding human disease.

We found a recessive *kfrs4* mutation in a KFRS4/Kyo strain that exhibits bilateral congenital cataract with progressive severe degeneration of the lens fibre cells. Using a positional cloning approach, we discovered a mutation in the major intrinsic protein of eye lens fibre gene (*Mip,* also known as aquaporin 0 or *Aqp0*), which is the most abundant membrane protein in the lens fibre cells, constituting more than 60% of the total membrane protein content of these cells [3], [4]. Spontaneous mutations in *Mip* (seven in humans and four in mice) have been associated with congenital cataract [5]–[14]. *Mip* mutant mice exhibit cataract as a result of disrupted lens differentiation [5], [7], [8], [15], [16]. These pathological observations suggest that MIP has essential roles in the establishment and maintenance of a uniform lens fibre structure and in fibre organisation.

All of the characterised *Mip* mutations are associated with cataract as the dominant phenotype, which could be explained by a specific dominant negative effect of the *Mip* mutant allele [17]–[19]. However, the cataract phenotype in KFRS4/Kyo rats is inherited in a recessive fashion, in contrast to known *Mip* mutations in humans and mice. In this study, we performed genetic, phenotypic and expression analyses of the KFRS4/Kyo rats. Our results suggest that this mutant should be classified as the first identified recessive mutant allele of *Mip.*

## Materials and Methods

### Rats

We used KFRS4/Kyo, BN/CrlCrlj, WIAR/lar and DOB/Oda rats as the wild-type rats in all of the experiments. KFRS4/Kyo and DOB/Oda rats were supplied by The National BioResource Project for the Rat in Japan (NBRP Rat: http://www.anim.med.kyoto-u.ac.jp/nbr/Default.aspx), Kyoto University (Kyoto, Japan). The BN/CrlCrlj and WIAR/Iar rats were purchased from Charles River Japan (Yokohama, Japan) and from the Institute for Animal Reproduction (Kasumigaura, Japan), respectively. All of the procedures involving animals met the guidelines described in the Proper Conduct of Animal Experiments, as defined by the Science Council of Japan, and were approved by the Animal Care and Use Committee of the Tokyo Metropolitan Institute of Medical Science, Tokyo University of Agriculture and Kyoto University.

### Phenotypic Analysis

The rat pupils were dilated using Mydrin-P (Santen Pharmaceutical, Osaka, Japan), and both eyes were observed after 5 min. The diagnosis of cataracts was performed by macroscopic examination, as described previously [20].

Both the right and left eyeballs from embryonic and postnatal wild-type, *kfrs4*/+ heterozygous and *kfrs4*/*kfrs4* homozygous rats were examined for histological analysis. The rats were sacrificed, and both eyeballs were enucleated and fixed in Superfix (Kurabo, Tokyo, Japan) overnight at room temperature. After fixation, specimens were transferred to methanol, dehydrated, embedded in paraffin, and sectioned (5 µm). After removing the paraffin, the sections were stained with haematoxylin and eosin and observed under a Leica DM2500 light microscope.

### Genetic Mapping

Genetic mapping of the *kfrs4* mutant locus was performed by intercrossing progeny derived from the mating of (KFRS4/Kyo × DOB/Oda) F_1_ × KFRS4/Kyo. The backcrossed progeny with a mutant phenotype were easily identified by the overt lens opacity induced by mydriatic instillation. DNA samples from 58 offspring, including 31 cataract-presenting rats of a KFRS4/Kyo and DOB/Oda cross, were genotyped using 108 polymorphic microsatellite markers selected from The NBRP Rat (Table S1) and six microsatellite markers (Table S2) developed from the rat genomic sequence (Ensembl: http://asia.ensembl.org/Rattus_norvegicus/Info/Index). Genotyping was carried out using PCR (Table S2) and 4% agarose gel electrophoresis. The map position was refined using the Map Manager computer program [21].

### Mutation Analysis

A genomic fragment covering the four coding exons of *Mip* was amplified from genomic DNA isolated from wild-type rats (DOB/Oda, BN/CrlCrlj and WIAR/Iar), *kfrs4*/+ heterozygous rats (F_1_ rats from a *kfrs4/kfrs4* and wild-type cross), and *kfrs4/kfrs4* homozygous rats. The primers Mip_F and Mip_R were used for amplification, and the following primers were used for sequencing: Mip_F1, Mip_F2, Mip_F3, Mip_F4, Mip_R1, and MIP_R2 (Table S2). The PCR products were purified using the QIAquick Gel Extraction Kit (Qiagen, Valencia, CA), sequenced using a BigDye Terminator kit (Life Technologies, Grand Island, NY) and analysed using an Applied Biosystems 3130×l Genetic Analyzer.

The *kfrs4* allele was genotyped using PCR to amplify genomic DNA and fluorescently labelled using Mip_del_F and Mip_del_R (Table S2). DNA samples from 20 inbred rat strains (ACI/NKyo, DON/Kyo, IS/Kyo, RCS/Kyo, SHR/Kyo, TM/Kyo, W/Kyo, WAG/Kyo, WTC/Kyo, ZI/Kyo, KDP/Tky, LE/Stm, F344/Stm, DOB/Oda, KFRS2/Kyo, KFRS3A/Kyo, KFRS3B/Kyo, KFRS4/Kyo, KFRS5A/Kyo, and KFRS6/Kyo) for control of genotyping were supplied by NBRP Rat. The PCR products were separated using a Beckman CEQ8000 instrument (Beckman Coulter, Fullerton, CA) with a size standard, and the fragment sizes were determined using fragment analysis software.

### RT-PCR

Total RNA was isolated from the eyes of 7-week-old wild-type and *kfrs4*/*kfrs4* rats using TRIzol (Life Technologies) and the TRIzol Plus Purification Kit (Life Technologies) according to the manufacturers’ protocols. The Superscript VILO cDNA synthesis kit (Life Technologies) was used to generate cDNA using 2 µg of DNase-pretreated total RNA. We also prepared cDNA from wild-type, *kfrs4*/+, and *kfrs4*/*kfrs4* rats at 7 and 10 weeks for quantitative RT-PCR (qRT-PCR). Primers for *Mip* and *Gapdh* were purchased from Qiagen (Valencia, CA). A total of 9 specific transcripts (*Cryaa*, *Crygb*, *Crygd*, *Casp6*, *Lim2*, *Bfsp1*, *Bfsp2*, *Gja3*, and *Gja8*) for lens fibre cells were used as the initial controls and to verify the quality of the fibre cells (Table S2). Subsequently, qRT-PCR was performed as previously described [20].

### Antibodies

An anti-MIP rabbit polyclonal antibody (MIP-Cter) that targets a 17-amino acid (aa) peptide in the C-terminal cytoplasmic domain of MIP was previously characterised [8], [22] and commercially acquired from Alpha Diagnostic International (San Antonio, TX). We also generated an anti-MIP rabbit polyclonal antibody (MIP-Nter) to a peptide (C+TPPAVRGNLALNT) in the N-terminal region of the MIP peptide from aa 108 to 120 (NM_001105719). We used N-cadherin (CDH2) and β-catenin (CTNNB1) as markers of the lens. Both proteins were evenly expressed in lens fibre cells [23]–[26]. Mouse monoclonal anti-CDH2 and anti-CTNNB1 antibodies were purchased from BD Biosciences (San Jose, CA). For a secondary antibody, Horseradish peroxidase (HRP)-conjugated donkey anti-rabbit and HRP-sheep anti-mouse IgG antibodies (GE Healthcare Life Science, Piscataway, NJ) were used for Western blotting. Alexa Fluor-conjugated secondary antibodies were obtained from Invitrogen.

### Immunoblotting

Membrane proteins from the eyes of wild-type, *kfrs4*/+, and *kfrs4*/*kfrs4* rats at 7 and 8 weeks of age were purified as described by Sidjanin et al. [7]. Samples containing approximately 500 ng of protein were separated on 15% SDS-polyacrylamide gels, and the separated proteins were transferred onto a Hybond-P PVDF membrane (GE Healthcare Life Science). MIP protein bands were detected using the anti-MIP-Cter (1∶1,000) and -Nter (1∶5,000) antibodies, followed by HRP-anti-rabbit (1∶20,000) IgG secondary antibodies. ECL Prime Western blotting detection regents (GE Healthcare Life Science) were used for the enhanced chemiluminescent detection of specifically bound antibody, and the membrane was exposed to autoradiography film (GE Healthcare Life Science). The membrane was then stripped and blotted with anti-CDH2 (1∶2,000) and CTNNB1 (1∶500) antibodies, followed by HRP-anti-mouse IgG secondary antibody (1∶20,000) as a protein loading control. For quantitative Western blot analyses, the chemiluminescently labelled bands were quantified using the ImageJ software (http://rsb.info.nih.gov/ij). The expression level in wild-type rats was assigned an arbitrary value of 1, and the differences were evaluated using one-way ANOVA with a post hoc Bonferroni multiple comparison test.

### Immunohistochemistry

Both the right and left eyes from wild-type (9, 11 and 14 weeks of age), *kfrs4*/+ (8, 9, 11, 13 and 15 weeks of age), and *kfrs4/kfrs4* (9 and 11 weeks of age) rats were used for immunostaining. The procedure for immunohistochemistry of lens paraffin sections was described previously [20], and the sections were stained with anti-MIP-Cter (1∶100), anti-MIP-Nter (1∶100), anti-CDH2 (1∶2,000) and anti-CTNNB1 (1∶500). Fluorescence images were obtained using a Zeiss LSM780 confocal microscope and processed using Adobe Photoshop software. For each immunofluorescence labelling and lens fibre cell quantification experiment, we used three images from three different section preparations. The fluorescence intensity of MIP was analysed using the Image J software to analyse confocal images that were taken under identical conditions and adjusted using the intensity of CDH2 and CTNNB1 as a control. The expression level in wild-type rats was assigned an arbitrary value of 1, and differences were evaluated using a two-tailed *t*-test.

## Results

### Isolation and Phenotypic Characterisation of the Spontaneous *kfrs4* Mutant

**Figure 1:**
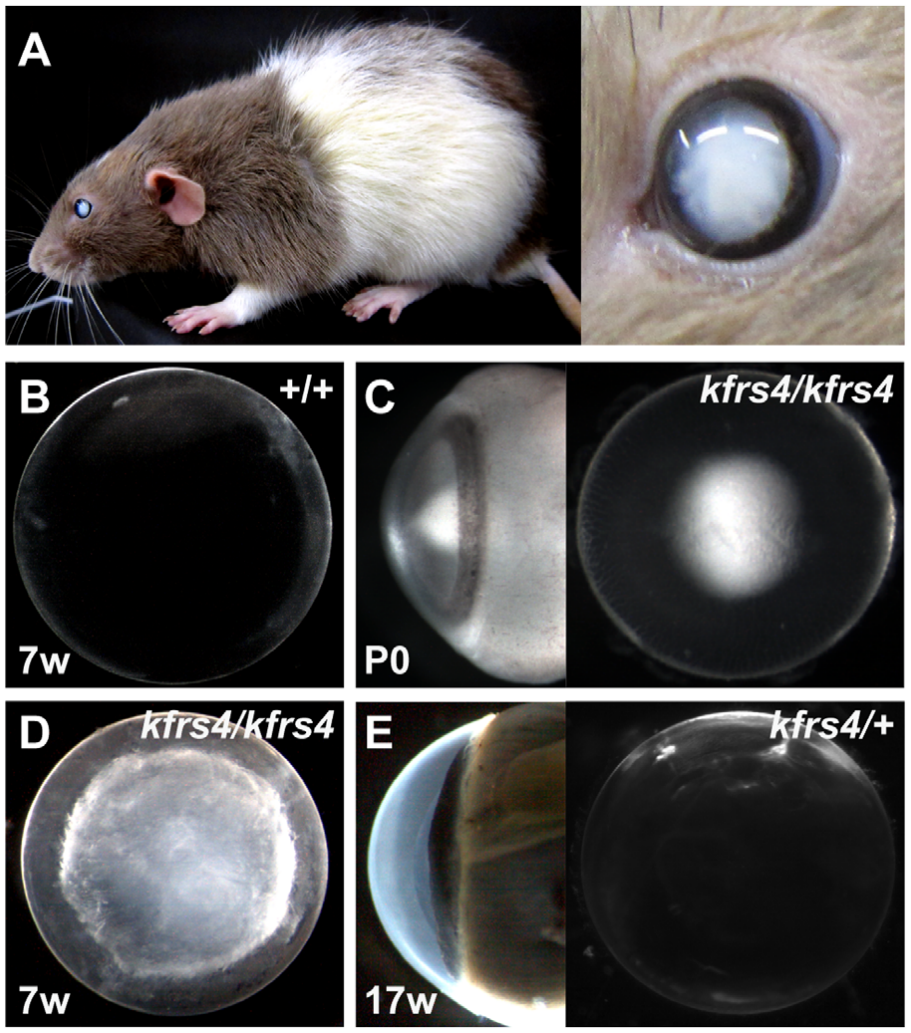
Lens opacities in *kfrs4* mutant rats. **A.** Gross appearance in *kfrs* rats at 7 weeks of age. Opacity was observed throughout the eye (right). **B–E.** Dark field imaging of the dissected lens from DOB/Oda (+/+, **B**), KFRS4/Kyo (*kfrs4/kfrs4*, **C, D**), and (KFRS4/Kyo × DOB/Oda) F_1_ (*kfrs4*/+, **E**) rats. Wild-type rats show normal transparency (**B**), but nuclear opacity is observed in the anterior (left) and side (right) views in a lens from a *kfrs4/kfrs4* homozygote at P0 (**C**). The *kfrs4/kfrs4* homozygous rat developed a dense nuclear cataract with full penetrance at 7 weeks of age (**D**). At 17 weeks of age, both *kfrs4*/+ heterozygotes and +/+ rats showed normal lens transparency (**E**).

While characterising rats from the KFRS4/Kyo strain, we observed that all of the rats developed severe bilateral lens opacity throughout the eye within 1 month of birth (Figure 1A). To classify the cataract type caused by the *kfrs4* mutation, lenses from wild-type and KFRS4/Kyo rats were dissected and observed under dark-field microscopy (Figure 1B-E). The opacity of the KFRS4/Kyo rat lenses was observed in the anterior nuclear regions at P0 (Figure 1C). The cataract progressed to a nuclear cataract and then to an all-over opacity that included the cortical fibres at 7 weeks of age (Figure 1D). The F_1_ progeny from the crossbreeding of DOB/Oda, BN/CrlCrlj, and WIAR/lar rats with KFRS4/Kyo rats showed normal lens phenotypes (Table 1). Crossbreeding between the (KFRS4/Kyo × DOB/Oda) F_1_ rats and KFRS4/Kyo rats produced 31 offspring with cataract and 27 offspring without cataract. Moreover, mating (KFRS4/Kyo × WIAR/lar) F_1_ rats with *kfrs4* rats produced 23 offspring with cataract and 29 offspring without cataract. These results indicate that the *kfrs4* mutation is recessive (Figure 1E, Table 1).

**Table 1:** Incidence of cataractogenesis in *kfrs4* F_1_ rats from crosses of KFRS4/Kyo and 3 inbred lines and in [(KFRS4/Kyo × DOB/Oda) F_1_ × KFRS4/Kyo] N_2_, and [(KFRS4/Kyo × WIAR/lar) F_1_ × KFRS4/Kyo] N_2_ rats.

			Phenotype		
Strain or cross	Age (W)	*n*	Cataract (*n*)	Normal (*n*)	Incidence (%)
KFRS4/Kyo	8	55	55	0	100
(KFRS4/Kyo × DOB/Oda) F_1_	12	33	0	33	0
	44	3	0	3	0
[(KFRS4/Kyo × DOB/Oda) F_1_ × KFRS4] N_2_	12	58	31	27	53.4
(KFRS4/Kyo × BN/CrlCrlj) F_1_	14	2	0	2	0
(KFRS4/Kyo × WIAR/Iar) F_1_	19	31	0	31	0
[(KFRS4/Kyo × WIAR/lar) F_1_ × KFRS4/Kyo] N_2_	12	52	23	29	44.2

**Figure 2:**
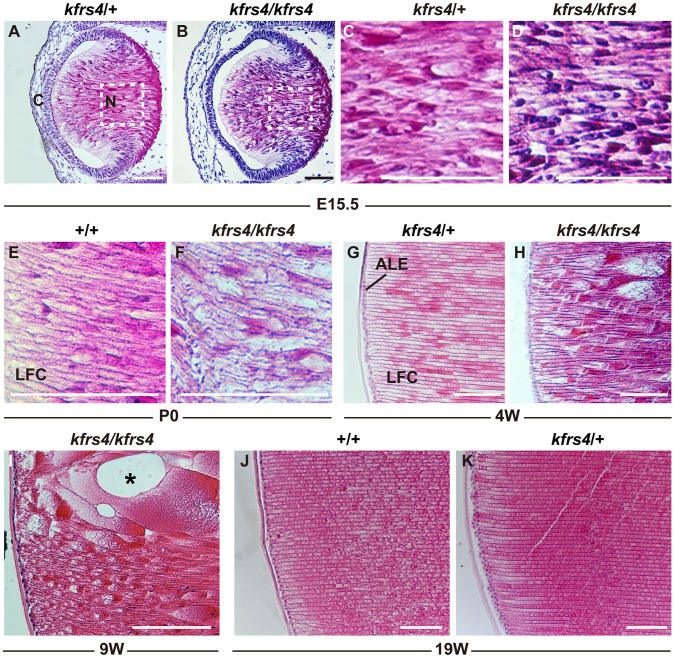
Lens phenotypes in +/+, *kfrs4*/+, and *kfrs4/kfrs4* rats at various embryonic and postnatal stages. Eyes were fixed, sectioned, and stained with haematoxylin and eosin. **A–D.** Lens histology in *kfrs4*/+ (**A, C**) and *kfrs4/kfrs4* (**B, D**) rats at E15.5. Highly magnified images of the dotted box area in **A** and **B** of the lens fibres in *kfrs4*/+ (**C**) and *kfrs4/kfrs4* (**D**) rats are shown. Co, cornea; N, nuclear region. **E–K.** Lens histology in +/+, *kfrs4*/+ and *kfrs4/kfrs4* rats at various postnatal stages. **E–F.** Comparison of phenotypes of the anterior fibres between +/+ (**E**) and *kfrs4/kfrs4* (**F**) rats at P0. LFC, lens fibre cell. **G–K.** Mature lens fibres in +/+ (**J**), *kfrs4*/+ (**G, K**) and *kfrs4/kfrs4* (**H, I**) rats. Large vacuoles (asterisk) were observed in the lens fibres of *kfrs4/kfrs4* rats at 9 weeks of age (**I**). ALE, anterior lens epithelium. Scale bar  = 200 µm.

To define the histological defects in the lenses of *kfrs4* mutants, sagittal sections of the eye were prepared from wild-type, *kfrs4*/+, and *kfrs4/kfrs4* rats at various embryonic and postnatal stages (Figure 2). At E15.5, the phenotypes of the lens in *kfrs4/kfrs4* homozygotes showed a pattern of development similar to that of *kfrs4*/+ heterozygotes (Figure 2A, B). However, there were signs of disorganisation in the fibre cells in the nuclear region of the *kfrs4/kfrs4* homozygous lens; the nuclei of the fibre cells remained in this region to a significant degree in comparison to the *kfrs4*/+ heterozygotes (Figure 2C, D). Degeneration of the lens fibre was pronounced in both the anterior and posterior regions in the *kfrs4/kfrs4* homozygotes at P0, with lens fibre swelling (Figure 2E, F). Over the next few weeks, the fibre cells became progressively more disorganised (Figure 2G, H), and irregularly shaped cells and huge vacuoles were present by 9 weeks of age (Figure 2I). In contrast, the lenses of *kfrs4*/+ heterozygotes exhibited compactly packed and uniform fibre cells at 19 weeks of age, similar to the lenses of wild-type rats (Figure 2J, K).

### Identification of the *kfrs4* Mutation

**Figure 3:**
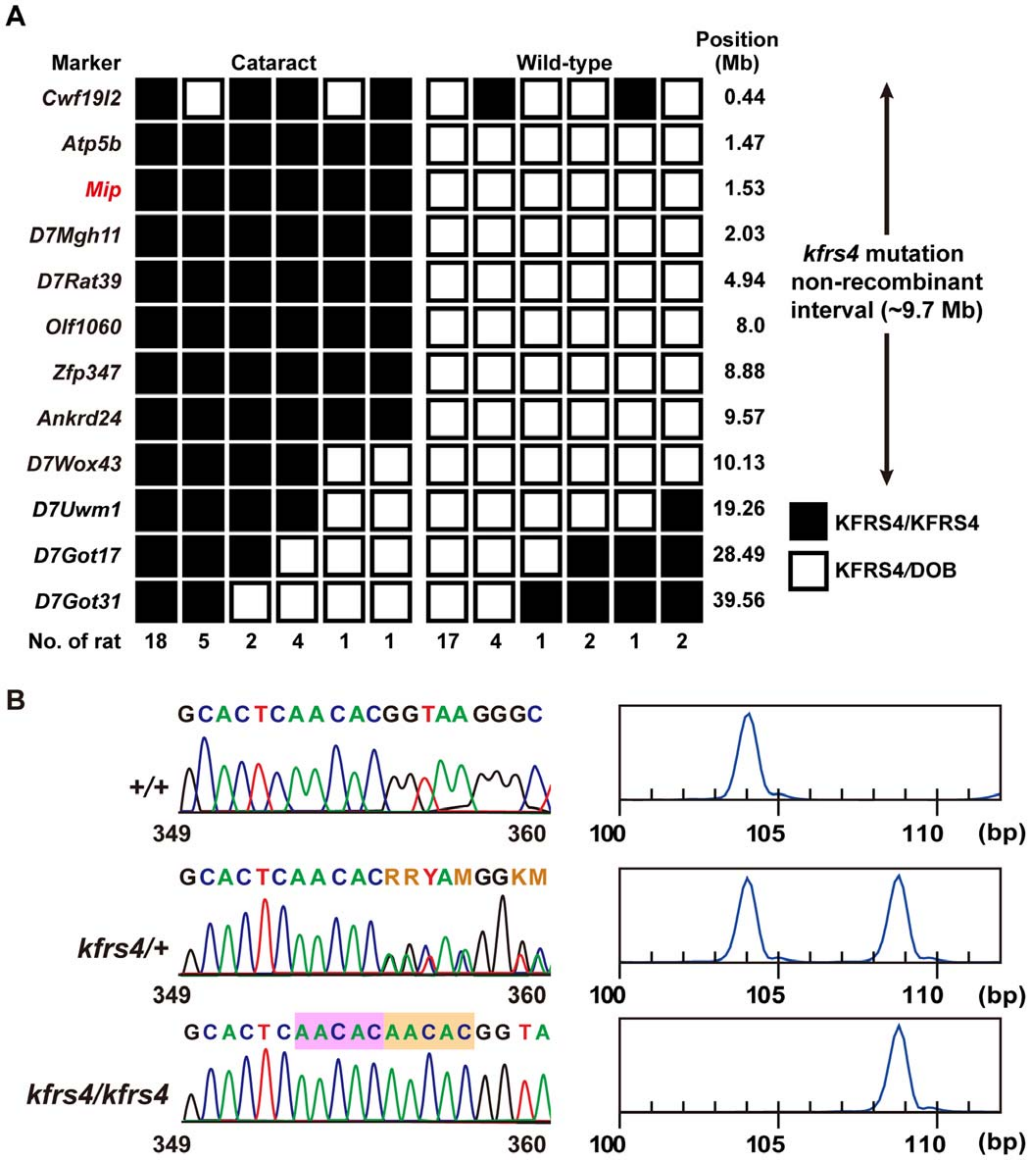
Positional cloning of the *kfrs4* mutation. **A.** Haplotype analysis for (KFRS4/Kyo × DOB/Oda) F_1_ × KFRS4/Kyo backcross progeny in the proximal region of chromosome 7. Markers are shown to the left. The number of offspring inheriting each type of chromosome is listed at the bottom of each column. **B.** Mutation analysis of *Mip* in the KFRS4/Kyo rat. Sequence analysis (left panels) for exon 1 of *Mip* in DOB/Oda (+/+, top), (KFRS4/Kyo x DOB/Oda) F_1_ (*kfrs4*/+, middle), KFRS4/Kyo (*kfrs4/kfrs4*, bottom) rats. A 5-bp insertion (AACAC, pink and dark orange labelled in the bottom panel) is found in *kfrs4*/+ and *kfrs4*/*kfrs4* rats. A size analysis (right panels) of fragments across the deletion revealed a 109-bp amplicon in the homozygous *kfrs4/kfrs4* rats (bottom) compared with a 104-bp amplicon in the +/+ rats (top); both amplicons were present in the *kfrs4*/+ rats (middle).

DNA samples from 58 [(KFRS4/Kyo × DOB/Oda) × KFRS4/Kyo] N_2_ progeny (Table 1) were genotyped using 114 microsatellite markers (Table S1) on rat chromosome 1–20 to determine the location of the *kfrs4* mutation. Using this linkage analysis, we mapped the *kfrs4* mutation to an interval of approximately 9.7-Mb between markers *Cwf19l2* and *D7Wox43* on chromosome 7 (Figure 3A). This region contains more than 350 protein coding genes, including the *kfrs4* candidate region [Ensembl]. Given its genomic locus and its involvement in a similar mutant pathology in humans and mice, *Mip* was the strongest candidate gene for the *kfrs4* mutation. The *kfrs4* phenotype was non-recombinant with a microsatellite marker in the 3′ region of *Mip* in 58 [(KFRS4/Kyo × DOB/Oda) ×KFRS4/Kyo] N_2_ progeny (Figure 3A).

By sequencing *Mip*, we identified a 5-bp insertion at nucleotide position 360 in exon 1 of *Mip* in the (KFRS4/Kyo × DOB/Oda) F_1_ and KFRS4/Kyo rats (Figure 3B). This 5-bp insertion, AACAC, is present as a tandem repeat, most likely resulting from the duplication of the same 5 bp 5′ of the insertion (Figure 3B). We designed a genotyping PCR primer pair to detect a 104-bp fragment for the wild-type *Mip* allele and a 109-bp fragment for the *kfrs4* allele. Based on these PCR amplicons, we were able to confirm the genotypes of the +/+, *kfrs4*/+, and *kfrs4/kfrs4* rats (Figure 3B). Moreover, the deletion was only observed in the *kfrs4* mutants and was not present among a set of 21 rat strains, including other KFRS strains (Figure S1).

**Figure 4:**
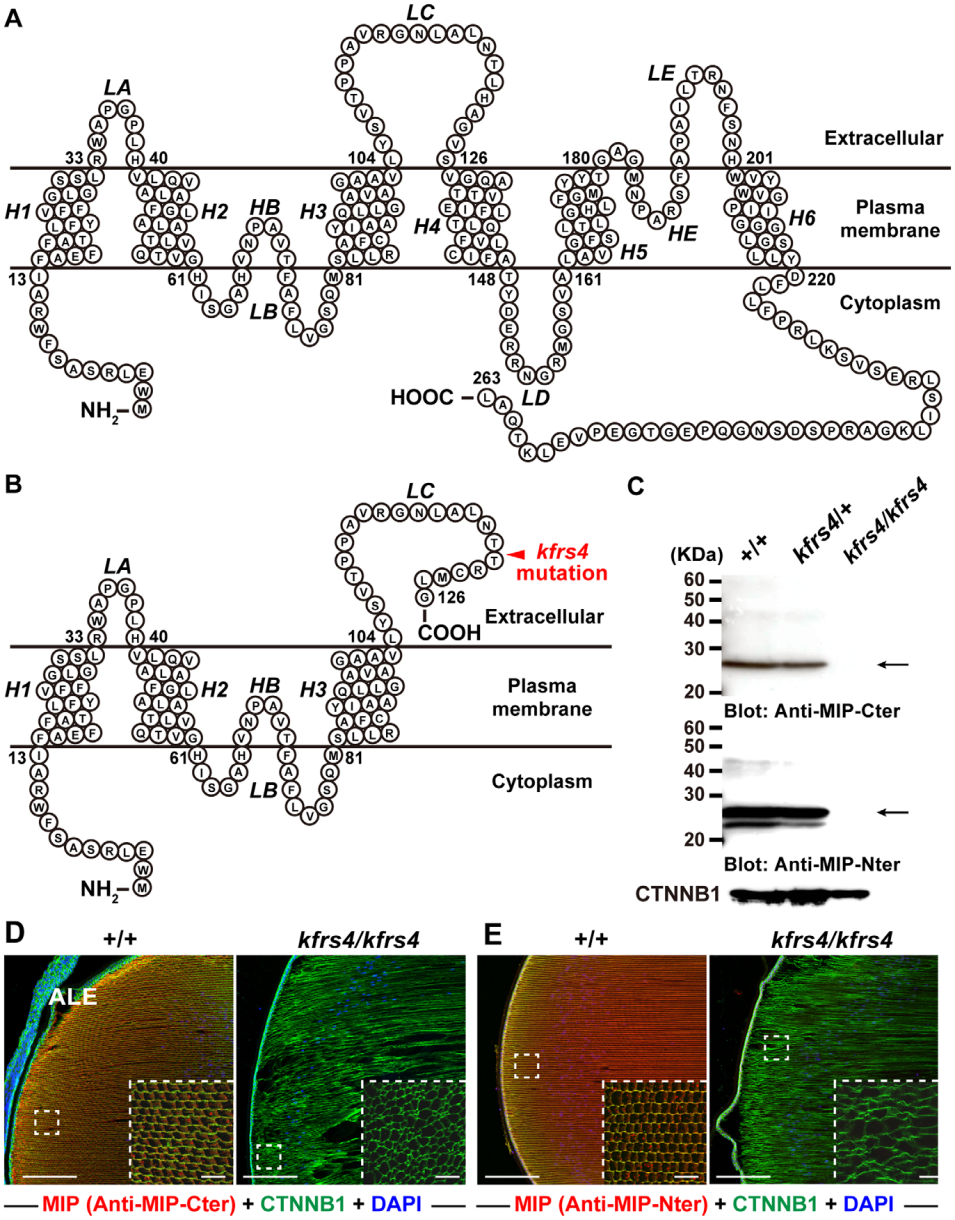
The *kfrs4* mutation produces a frameshift and the generation of a C-terminally truncated MIP protein. **A, B.** Predicted structures of the MIP proteins encoded by the wild-type (**A**) and *kfrs4* mutant (**B**) alleles. A schematic diagram showing the presumed membrane topology of MIP (modified from Francis et al. [17]). The locations of the six transmembrane domains (*H1, H2, H3, H4, H5,* and *H6*), two hemichannels (*HB* and *HE*), and the extracellular (*LA, LC,* and *LE*) and intracellular (*LB* and *LD*) loops are indicated. The schematic illustrates premature truncation of the protein expressed from the *Mip^kfrs4^* allele as a result of the mutation (**B**). **C.** Western blots of homogenates prepared from the eyes of +/+, *kfrs4/*+ and *kfrs4/kfrs4* rats detected with anti-MIP-Cter (top) and anti-MIP-Nter (middle) antibodies. Note the absence of a specific band at ∼28 kDa (arrows) in the *kfrs4/kfrs4* mutant homogenates. The samples were processed for indirect immunofluorescence using an anti-CNTTB1 antibody (bottom). **D,** **E.** Immunofluorescence labelling of MIP in the lens of +/+ (left panels) and *kfrs4/kfrs4* (right panels) rats at 7 weeks of age. These panels show merged images of the detection of MIP (red)+CNTTB1 (green)+DAPI (blue). The small panels within the large panels show higher magnifications of the dashed boxed areas in the large panels. ALE, anterior lens epithelium. Scale bars  = 200 µm (large panels) and 50 µm (small panels).

The *kfrs4* 5-bp insertion is predicted to cause a frameshift mutation that results in truncation of the peptide chain by generating a stop codon at amino acid position 127, and this *kfrs4* frameshift mutation causes a truncation of the MIP protein that removes three transmembrane domains (*H4, H5* and *H6*), the *HE* hemichannels, cytoplasmic loop D, and extracellular loop E (Figure 4A, B). To confirm this truncation of the C-terminal domain in *kfrs4* rats, we performed immunoblot and immunohistochemistry analyses using a rabbit polyclonal antibody, anti-MIP-Cter, that targets a 17-aa peptide in the C-terminal cytoplasmic domain of MIP [Alpha Diagnostic International]. The 28-kDa (previously reported as the MIP band [27]) bands were abundant in extracts from the wild-type and *kfrs4*/+ heterozygote eyes but were not detectable in the eye extracts from *kfrs4* homozygotes (Figure 4C). Moreover, we generated an antibody, anti-MIP-Nter, to a peptide within an LC extracellular domain in the N-terminal region of the *kfrs4* mutation to detect the presence of the mutant MIP protein in *kfrs4* mutants. In the eye extracts of wild-type and *kfrs4*/+ rats, this antibody, as well as anti-MIP-Cter antibody, detected the 28-kDa band (Figure 4C). In addition, we detected a variant of approximately 23 kDa. Although we have not yet identified whether these bands represent nonspecific antibody binding or a degradation product, these bands could not be detected in the eye extracts of *kfrs4*/*kfrs4* homozygotes.

As reported previously [27], MIP was expressed throughout the fibre cells of the lens in wild-type rats; we also observed strong signals in the anterior fibre cells of adult rats (Figure 4D). The MIP staining was abolished in the *kfrs4* homozygous rats, confirming the truncation of the C-terminal domain in rats with the *kfrs4* mutation (Figure 4D). Immunohistochemistry using the anti-MIP-Nter antibody revealed a nearly identical staining pattern to that of anti-MIP-Cter (Figure 4E). We found that the staining of MIP is completely ablated in the *kfrs4*/*kfrs4* homozygotes.

### Reduction in *Mip* mRNA and MIP Protein Expression by the *kfrs4* Mutation

**Figure 5:**
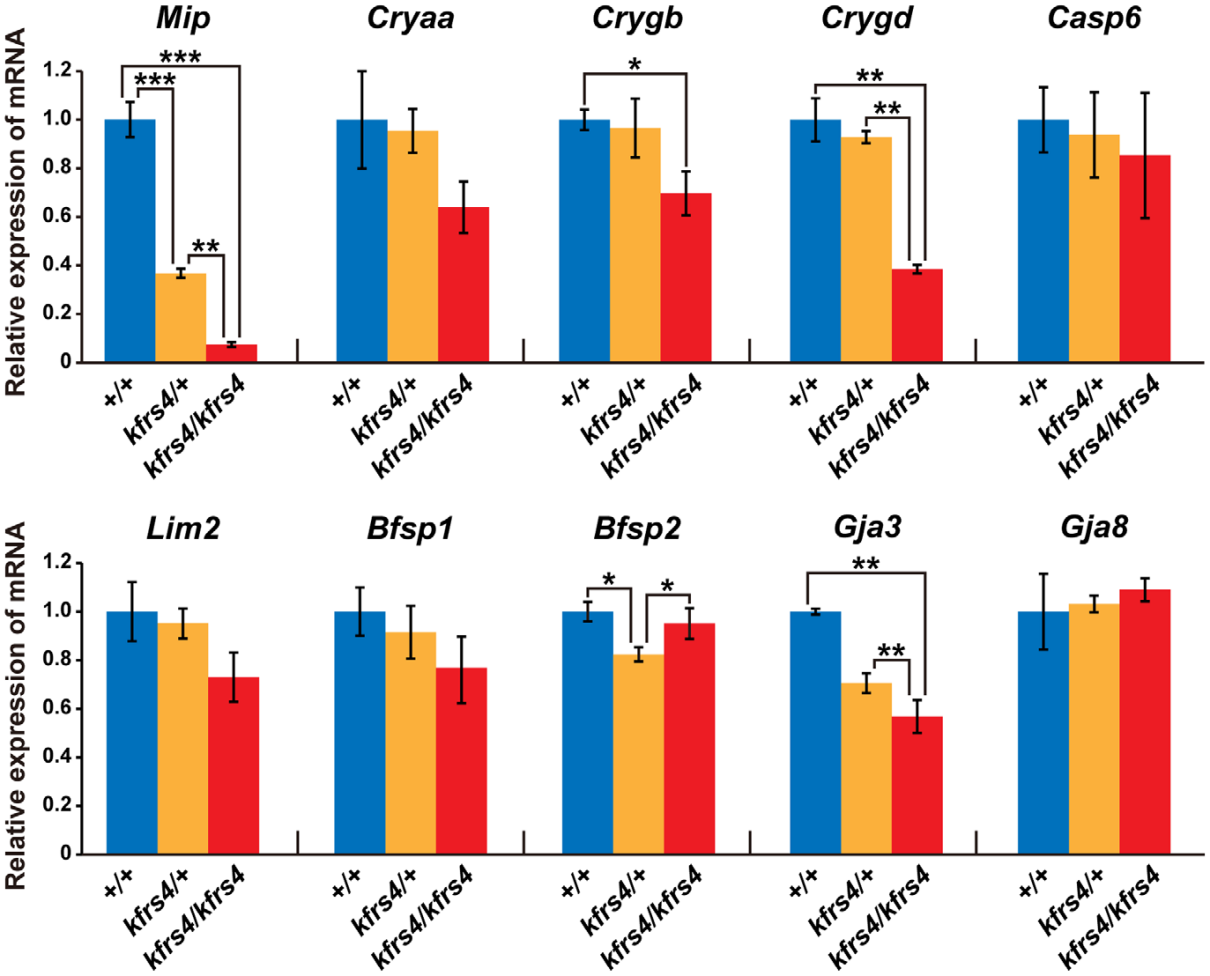
Quantitative analysis of selected lens fibre specific gene expression in +/+, *kfrs4*/+, and *kfrs4/kfrs4* rats. **A.** Relative levels of mRNA in the lenses of +/+ (blue bars), *kfrs4*/+ (orange bars), and *kfrs4/kfrs4* (red bars) rats at 7 weeks of age. mRNA expression was detected using real-time quantitative RT-PCR analysis. *P<0.05, **P<0.01, ***P<0.001.

As mentioned above, the frameshift in the *kfrs4* mutant produces a truncated MIP protein that is missing several domains in the C-terminal region (Figure 4). To examine the effect of the *kfrs4* mutation on *Mip* mRNA expression, we carried out real-time qRT-PCR using RNA from wild-type, *kfrs4*/+ heterozygous, and *kfrs/kfrs4* homozygous rats. The relative abundance of *Mip* transcripts in the eyes of *kfrs4*/+ and *kfrs/kfrs4* rats was approximately 34.6 and 7.1% of wild-type levels, respectively (Figure 5). To confirm this reduction of *Mip* mRNA in *kfrs4*/+ and *kfrs/kfrs4* eyes and determine whether it caused a loss of lens fibre cells to induce cataracts, we performed qRT-PCR analysis of nine other lens-specific transcripts. Five of these transcripts, *Casp6, Lim2, Bfsp1,* and *Gja8*, did not exhibit significant expression changes among the wild-type, *kfrs4*/+, and *kfrs/kfrs4* rats (Figure 5). The expression of three crystallins, *Cryaa, Crygd,* and *Crygd*, exhibited similar levels between the wild-type rats and the *kfrs4*/+ heterozygous rats, but significant reductions were detected in the *kfrs/kfrs4* homozygous rats, suggesting the possibility that the reduction of these crystallins caused structural defects in the lens fibre cells of *kfrs/kfrs4* homozygous rats. Although the expression of *Bfsp2* (a code lens structural protein) was lower in *kfrs4*/+ rats relative to wild-type, a change in expression was not detected in the *kfrs/kfrs4* rats (Figure 5). These results may confirmed that the reduction of *Mip* expression in *kfrs4*/+ and *kfrs4/kfrs4* rats is not the result of cataract formation but is instead caused by the expression of certain genes that were affected in the *kfrs4* mutant. *Gja3* (also known as connexin 46) showed a significantly lower expression in *kfrs4*/+ and *kfrs/kfrs4* rats compared with wild-type (Figure 5). Although we could not explain why the expression of *Gja3* is also reduced in *kfrs/kfrs4* homozygous rats, the GJA3 protein directly interacts with MIP, mediated by the C-terminal region [28]; therefore, it may be an effect of the reduction in *Mip* mRNA as a result of the *kfrs4* mutation.

**Figure 6:**
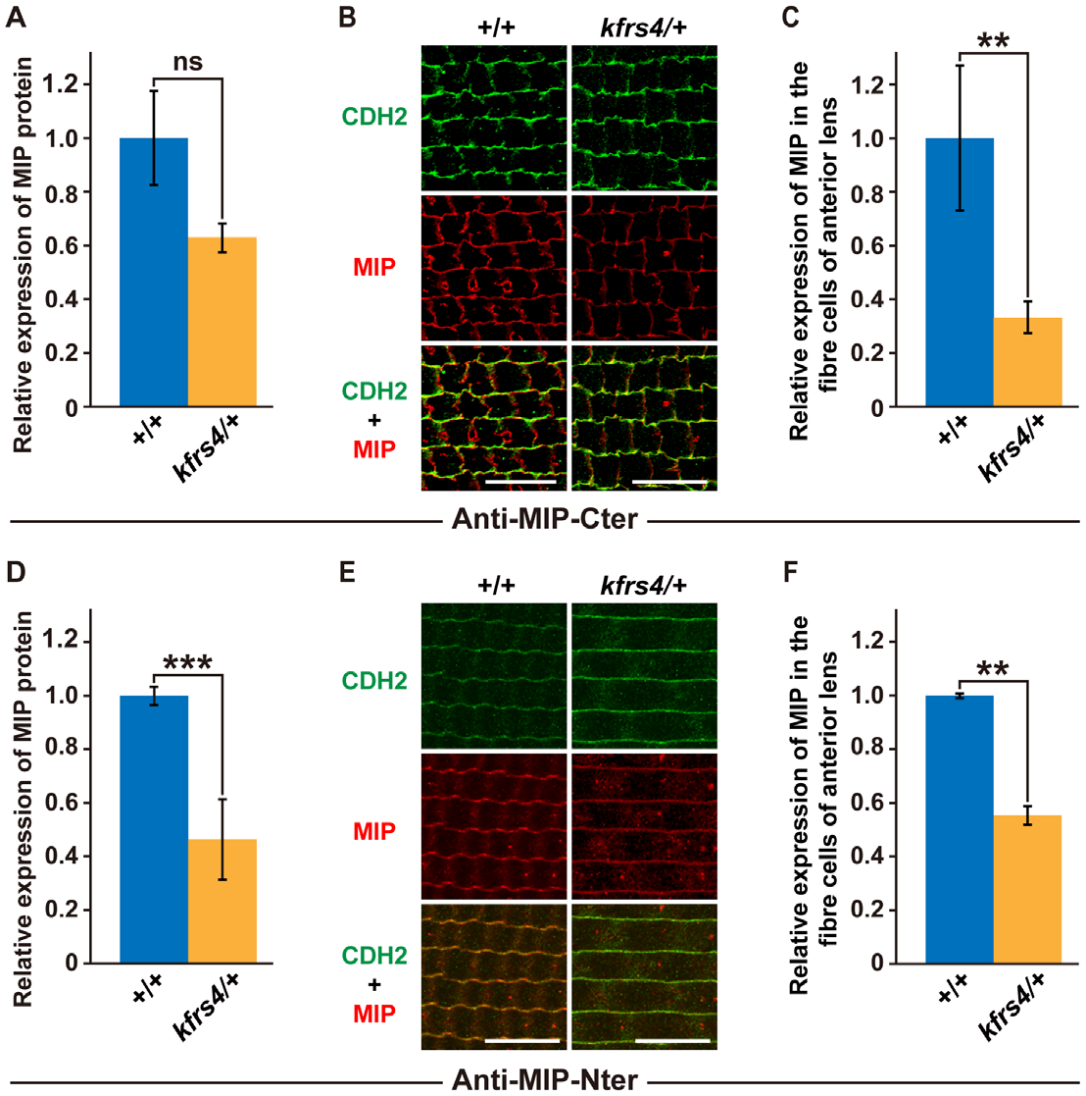
Quantitative analysis of MIP protein expression in lenses from +/+ and *kfrs4*/+ rats. A, D. Densitometric quantification of MIP expression levels detected by Western blot analysis using the anti-MIP-Cter (**A**) and anti-MIP-Nter (**D**) antibodies in the eyes of +/+ and *kfrs4*/+ rats at 7 weeks of age. **B, E.** Immunofluorescence labelling of CDH2 (top), MIP (middle), and merged images (bottom) in the lens fibres from +/+ (left) and *kfrs4*/+ (right) rats at 8 weeks of age. The sections are stained by both anti-MIP-Cter (**B**) and anti-MIP-Nter (**E**) antibodies. Scale bar  = 20 µm. **C, F.** Quantification of MIP intensities in **B** and **E**. The values shown in each graph (**A**, **C, D,** and **F**) indicate the mean relative expression levels and the standard errors of triplicate samples (*n*  = 3). **P<0.01, ***P<0.001, n.s., not significant.

Quantitative immunoblotting and immunohistochemistry were also performed to confirm the MIP protein expression levels in wild-type and *kfrs4*/+ heterozygous rats. Although the difference in protein levels was not significant according to a statistical analysis, MIP was 1.59 times less abundant in *kfrs4*/+ eyes than in wild-type eyes (Figure 6A). Quantitative immunohistochemical analysis of MIP in the fibre cells of the anterior lens revealed that the expression level of MIP significantly decreased in the *kfrs4*/+ rat, whereas the level of CDH2 did not decrease (Figure 6B). As shown in Figure 6C, the amount of MIP in *kfrs4*/+ eyes was 33.1% of the level observed in wild-type eyes. Similar results were obtained using the anti-MIP-Nter antibody and CTNNB1 as control for quantitative analysis (Figure 6D–F, Figure S2).

## Discussion

We present several lines of evidence demonstrating that a mutation in the *Mip* gene underlies congenital cataract in *kfrs4* mutant rats. First, *Mip* is located within the candidate region identified for the *kfrs4* mutation (Figure 3A). Second, the *kfrs4* mutant phenotypes were consistent with the *Mip* mutation genotypes in all of the rats examined (Figure 3B, Figure S1). Third, the absence of MIP-positive bands in the Western blots of eye tissue from *kfrs4* mutants and the lack of MIP-specific immunofluorescence in eye sections from *kfrs4* mutants indicate the absence of the normal *Mip* gene product (Figure 4B–G). The identified mutation is a 5-bp nucleotide insertion within the coding region of the amino terminus of MIP, adding to the catalogue of known *Mip* mutations that cause cataract in mammals.

In humans and in mice, point mutations [5], [6], [10]–[14], intragenic in-frame deletions [7], [8] and frameshift mutations [5], [9] in *Mip* have been characterised to cause dominant phenotypes (Figure S3). The cataract phenotype in *Mip^Hfi/+^* heterozygous mice suggested that a 76-bp deletion in *Mip* is a gain-of-function mutation, consistent with data showing that a normal level of wild-type MIP protein expression observed in *Mip^Hfi/+^*could not protect the lens from cataract formation [7]. Another *Mip* mutation in mice, *Mip^Cat-Tohm^* is a 12-bp deletion in *Mip* that does not alter the open reading frame, but these mutant mice exhibited a more severe cataract phenotype than did *Mip* knockout mice [15], [16] with a null mutation in *Mip* [8]. Interestingly, the lens fibre cells of a transgenic mouse that expressed both the wild-type and the *Mip^Cat-Tohm^* mutated *Mip* showed more severe degeneration at birth than did the lens cells of *Mip^Cat-Tohm/Cat-Tohm^* homozygotes, which expressed only *Mip^Cat-Tohm^.* In addition, the E134G/T138R mutant of human *MIP* leads to congenital cataract and results in a loss of water permeability owing to a failure in protein trafficking to the plasma membrane in homozygotes [6]; however, when the E134G/T138R mutant is co-expressed with wild-type MIP protein, the mutant protein reaches the plasma membrane but causes tetramer instability and a loss-of-function of wild-type MIP [17]. Moreover, FRET analysis of human *Mip^ADC2^* and wild-type MIP coexpressed in mammalian cells demonstrated that this mutation is dominant because the hetero-oligomerisation of the wild-type and mutant MIP molecules traps the wild-type MIP in the endoplasmic reticulum [18]. These studies indicate that mutated versions of the MIP protein have a strong dominant-negative effect on lens transparency and that cataract in these mouse and human cases is caused by gain-of-function mutations. However, we characterised *kfrs4* as a recessive mutation because *kfrs4/*+ heterozygous rats exhibit a lens structure that is similar to that of wild-type rats until the late stages of development (Figure 1, 2, Table 1), and the *kfrs4* mutation was effectively mapped in a linkage study using backcrosses to follow the recessive phenotype (Figure 3A). The *kfrs4/*+ heterozygous and *kfrs4/kfrs4* homozygous rats appear to have reduced *Mip* mRNA expression (Figure 5). Moreover, the mutant MIP protein in *kfrs4* rats presumably lacks 136 aa from the C-terminus because MIP signals were not detected by Western blotting and immunohistochemistry when an anti-MIP-Cter antibody that recognise C-terminal region of MIP was used (Figure 4, 6). These results suggest that the frameshift caused by the *kfrs4* mutation leads to functional inactivation though the rapid degradation of mRNA by nonsense-mediated mRNA decay; this observation supports the characterisation of *kfrs4* as a loss-of-function mutation in *Mip,* which may be explained by a partial loss of MIP function via dosage effects. Indeed, *Mip* null mutations may be characterised as semi-dominant because heterozygous mutants present with a milder cataract phenotype than do homozygotes [22], [27]. In addition, *Mip^Cat-Ft^* ^/+^ (in which aa 203–263 at the C-terminus has been replaced with a transposon sequence) and *Mip^Lop^* ^/+^ (A51P) heterozygous mutants also show mild phenotypes compared with the corresponding homozygous mutants [5]. Furthermore, the phenotype can be rescued in chimeric mice, which have lenses containing equal numbers of mutant and wild-type fibre cells [29].

Finally, the difference in heterozygous phenotypes and the inheritance mode in *kfrs4* rats may result from the diversity of the genetic background and species differences between rats and mice compared to humans, but we lack the evidence to make this determination. In humans, individuals with *MIP* mutations have disorders of multiple genetic origins that exhibit marked phenotypic heterogeneity [6], [10]–[14], [30]. In particular, it is known that cataracts caused by different types of mutant MIP have different phenotypes, suggesting that *Mip* mutations cause phenotypic heterogeneity [6]. The diverse phenotypes exhibited by the *Mip* gene suggest that other genetic modifiers are likely to influence the expression and function of MIP in lens development and in lens fibre formation.

## Supporting Information

Figure S1 **Gain of the 5-bp insertion in exon 1 of** ***Mip*** **in a KFRS4/Kyo strain.** Genotyping of various wild-type strains of rat, including other KFRS strains (asterisks), revealed an absence of the 5-bp insertion.
(TIF)

Figure S2 **Quantitative analysis of MIP protein expression in lenses from +/+ and** ***kfrs4*** **/+ rats.** **A, D.** Densitometric quantification of MIP expression levels detected by Western blot analysis using the anti-MIP-Cter (**A**) and anti-MIP-Nter (**D**) antibodies in the eyes of +/+ and *kfrs4*/+ rats at 7 weeks of age. **B, E.** Immunofluorescence labelling of CTNNB1 (top), MIP (middle), and merged images (bottom) in the lens fibres from +/+ (left) and *kfrs4*/+ (right) rats at 8 weeks of age. The sections are stained by both anti-MIP-Cter (**B**) and anti-MIP-Nter (**E**) antibodies. Scale bar  = 20 µm. **C, F.** Quantification of MIP intensities in **B** and **E**. The values shown in each graph (**A**, **C, D,** and **F**) indicate the mean relative expression levels and the standard errors of triplicate samples (*n*  = 3). **P<0.01, n.s., not significant.
(TIF)

Figure S3 **Alignment of MIP amino acid sequences and mutations in** ***Mip*** **responsible for congenital cataract in humans, mice and rats.** Amino acid sequences of human MIP (GenBank accession no. NM_012064) identical to those of mouse (NM_008600) and rat MIP (NM_001105719) are shown as dots. The numbers at the top of the human sequence indicate nucleotide positions. The locations of the six transmembrane domains (*H1, H2, H3, H4, H5,* and *H6*) and two hemichannels (*HB* and *HE*) are indicated by underlines and dotted underlines, respectively. The locations of the extracellular loops (Loop A, C, and E) and intracellular loops (Loop B and D) are indicated between the corresponding transmembrane domains. Eight reported human mutations, four reported mouse mutations, and the *kfrs4* rat mutation are indicated in green, blue, and red, respectively.
(TIF)

Table S1 **Microsatellite markers used in genetic mapping of the rat** ***kfrs4*** **locus.**
(XLSX)

Table S2 **Primers and PCR conditions used for the genetic mapping, mutation analysis, and qPCR in this study.**
(XLSX)
