# Supplementary figures and images for: A 5-bp Insertion in Mip Causes Recessive Congenital Cataract in KFRS4/Kyo Rats
Source: PLoS One. 2012 Nov 30;7(11):e50737. doi: 10.1371/journal.pone.0050737 (PMC3511373; doi:10.1371/journal.pone.0050737)

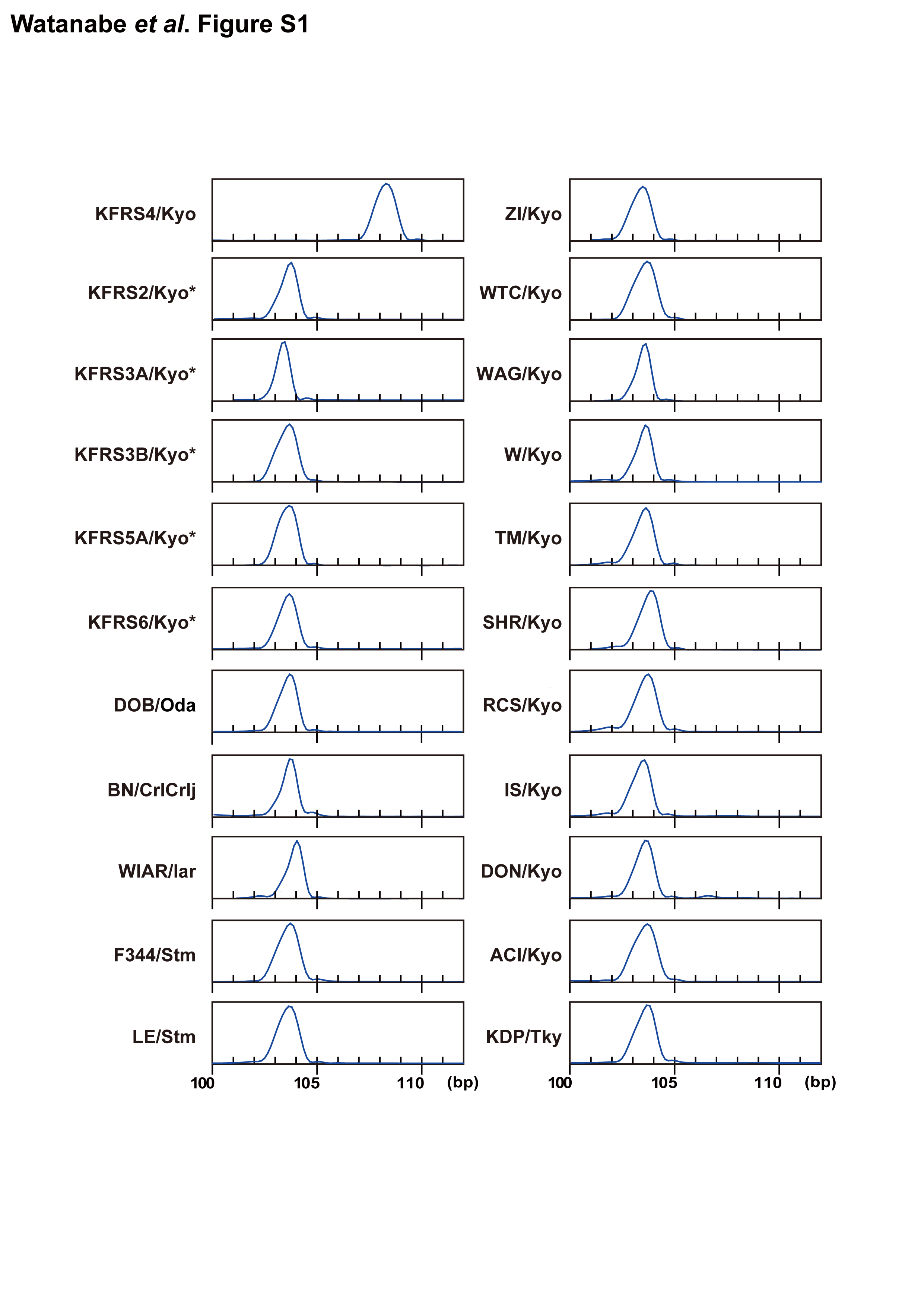

Supplement: Figure S1 — Gain of the 5-bp insertion in exon 1 of Mip in a KFRS4/Kyo strain. Genotyping of various wild-type strains of rat, including other KFRS strains (asterisks), revealed an absence of the 5-bp insertion. (TIF) [file pone.0050737.s001.tif]

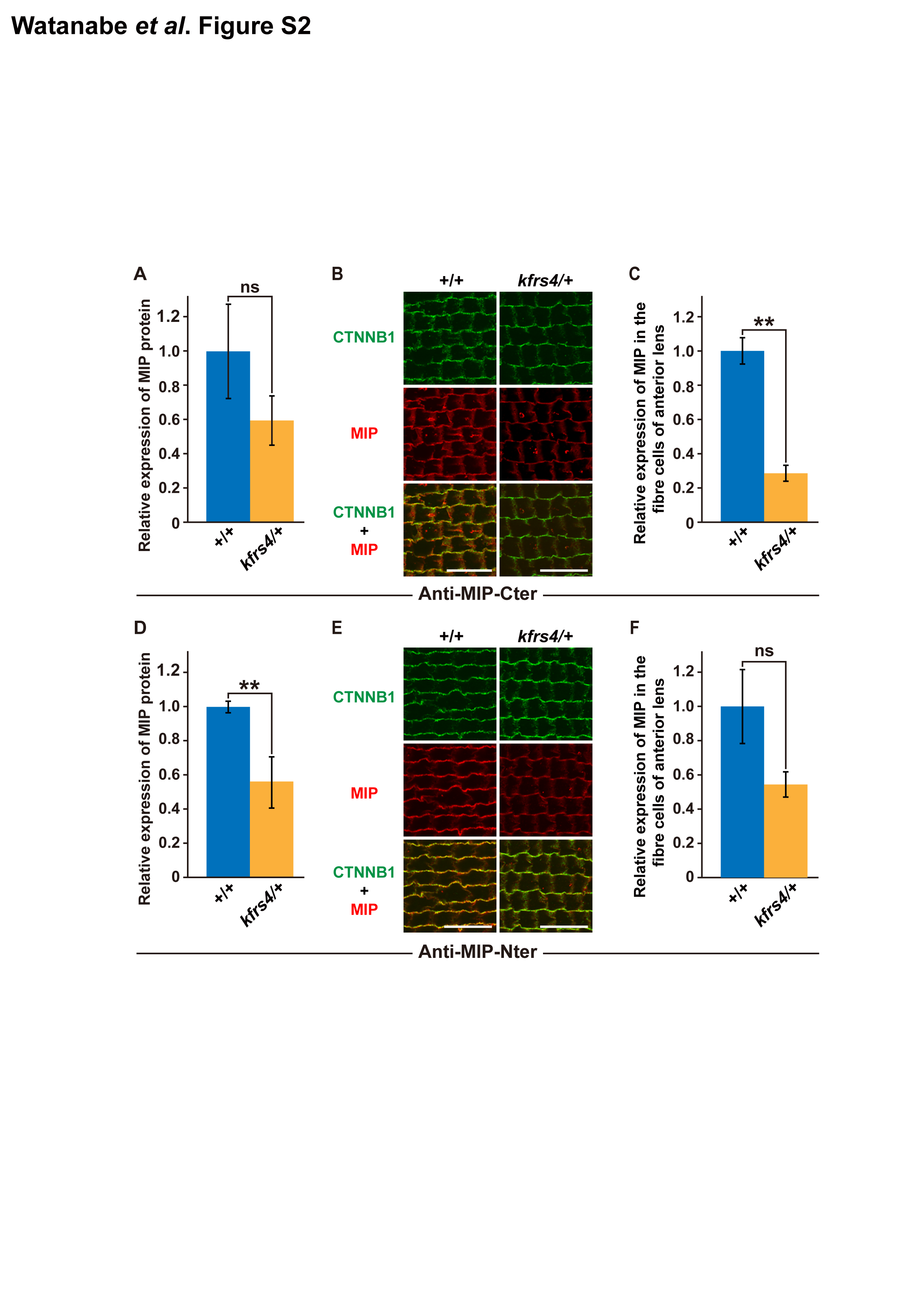

Supplement: Figure S2 — Quantitative analysis of MIP protein expression in lenses from +/+ and kfrs4 /+ rats. A, D. Densitometric quantification of MIP expression levels detected by Western blot analysis using the anti-MIP-Cter (A) and anti-MIP-Nter (D) antibodies in the eyes of +/+ and kfrs4/+ rats at 7 weeks of age. B, E. Immunofluorescence labelling of CTNNB1 (top), MIP (middle), and merged images (bottom) in the lens fibres from +/+ (left) and kfrs4/+ (right) rats at 8 weeks of age. The sections are stained by both anti-MIP-Cter (B) and anti-MIP-Nter (E) antibodies. Scale bar = 20 µm. C, F. Quantification of MIP intensities in B and E. The values shown in each graph (A, C, D, and F) indicate the mean relative expression levels and the standard errors of triplicate samples (n = 3). **P<0.01, n.s., not significant. (TIF) [file pone.0050737.s002.tif]

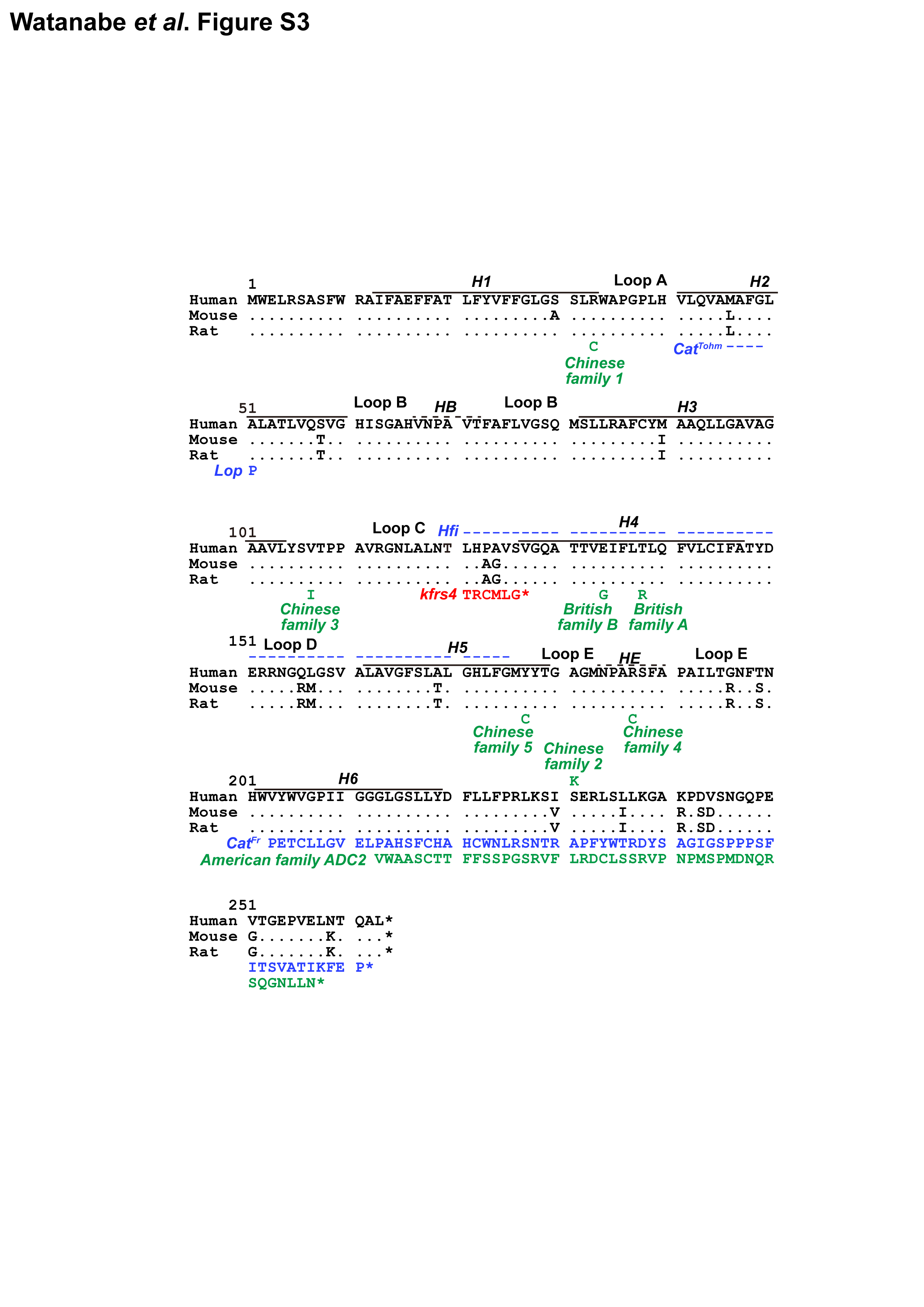

Supplement: Figure S3 — Alignment of MIP amino acid sequences and mutations in Mip responsible for congenital cataract in humans, mice and rats. Amino acid sequences of human MIP (GenBank accession no. NM_012064) identical to those of mouse (NM_008600) and rat MIP (NM_001105719) are shown as dots. The numbers at the top of the human sequence indicate nucleotide positions. The locations of the six transmembrane domains (H1, H2, H3, H4, H5, and H6) and two hemichannels (HB and HE) are indicated by underlines and dotted underlines, respectively. The locations of the extracellular loops (Loop A, C, and E) and intracellular loops (Loop B and D) are indicated between the corresponding transmembrane domains. Eight reported human mutations, four reported mouse mutations, and the kfrs4 rat mutation are indicated in green, blue, and red, respectively. (TIF) [file pone.0050737.s003.tif]
